# Investigation of Minerals Extracted during Seawater Desalination Using Two-Dimensional Correlation Spectroscopy

**DOI:** 10.3390/molecules28237852

**Published:** 2023-11-29

**Authors:** Ho Ji, Yeonju Park, Young Mee Jung

**Affiliations:** 1 Seawater Utilization Plant Research Center (SUPRC), Korea Research Institute of Ships & Ocean Engineering, Goseong-gun 24747, Republic of Korea; 79hoji@kriso.re.kr; 2 Department of Chemistry, Institute for Molecular Science and Fusion Technology, Kangwon Radiation Convergence Research Support Center, Kangwon National University, Chuncheon 24341, Republic of Korea

**Keywords:** desalination, spectroscopy, seawater, mineral, 2D-COS

## Abstract

In this study, mineral components extracted during the desalination process were concentrated and dried, and then identified using energy dispersive X-ray spectroscopy (EDS), X-ray diffraction (XRD), infrared (IR), and Raman spectroscopy. For detailed identification, two-dimensional correlation spectroscopy (2D-COS) was also applied to the XRD patterns, IR spectra, and Raman spectra of the minerals obtained from each desalination step. The EDS results confirm the presence of seawater minerals rich in Na^+^ ions in the first and second extracts, Ca^2+^ ions are present only in these stages, and Mg^2+^ ions are abundant in the third and final extracts. The presence of NaCl and MgSO_4_ minerals in the first to third and final extracts, respectively, was confirmed using XRD patterns. From the IR and Raman spectra, we found that the degree of hydration of SO_4_^2−^-related extracts decreased as seawater underwent desalination. Furthermore, 2D-COS provides information about the changes in the extracts obtained from the first to final stage. Heterospectral XRD and Raman 2D-COS provides clear assignments for Raman spectra. The use of 2D-COS helps to understand the characteristics of seawater extracts during the desalination process, and provides a better understanding of chemical and structural adaptations within the extract. As a result, this method contributes to an improved understanding of the desalination process and final products.

## 1. Introduction

Deep ocean minerals exist as various types of naturally occurring inorganic solid materials with specific chemical compositions and crystalline structures. Among several types of minerals in the deep ocean, polymetallic nodules, cobalt-rich ferromanganese crusts, polymetallic sulfides, and methane hydrates are of particular interest. This is because they can have economic value due to their metal content and potential use in various industries, including technology and energy. Additionally, investigation of these minerals can provide insights into geological and environmental processes in the deep ocean [1,2].

In seawater desalination, it is very important to remove some minerals, such as salt, to produce fresh water suitable for various applications, such as drinking, irrigation, and industrial processes [3,4,5]. In seawater desalination, the elimination of minerals is a critical step in the process. Two methods are commonly used for eliminating minerals: reverse osmosis (RO) and distillation [6,7,8]. RO is one of the most widely used methods for desalination and involves the use of high-pressure pumps and membranes. In this process, a high-pressure pump provides the energy needed to overcome the osmotic pressure of seawater, allowing free water to permeate through the membrane. In the RO method, seawater is pressurized and forced through a semipermeable membrane that allows water molecules to pass through while blocking salts and minerals. The concentrated brine, containing the extracted salts and minerals, is separated from the fresh water and released back into the ocean in accordance with environmental regulations. This method is effective in eliminating most of the dissolved minerals from seawater, producing high-quality fresh water. However, it has drawbacks in large-scale desalination due to high seasonal variations and varying progress of membrane fouling during long-term filtration. There are two distillation processes for large-scale facilities: multi-effect distillation (MED) and multi-stage flash distillation (MSF) [6,9,10]. These desalination techniques involve heating seawater to produce water vapor, which is then condensed into fresh water. The process eliminates minerals through the separation of liquid water from dissolved salts. In MDE, multiple stages with decreasing pressures are used to evaporate water at different temperatures. In MSF, seawater is flash-evaporated at different pressures and temperatures to produce water vapor. It can be used in large-scale systems, but is generally less energy efficient than RO. While these desalination methods effectively eliminate minerals, importantly, they produce a concentrated brine byproduct containing the extracted minerals. To reduce its environmental impacts, proper disposal or treatment of this brine is essential.

Identification of the minerals in desalinated water from concentrated seawater is essential to assess the composition of the byproducts, to understand possible scaling or fouling, and to manage the disposal of seawater concentrate in an environmentally responsible manner. There are various methods of mineral identification, including ion chromatography (IC) [11,12], inductively coupled plasma spectrometry (ICP) [13,14,15], X-ray diffraction (XRD) [13,16], scanning electron microscopy (SEM) [13,17], energy-dispersive X-ray spectroscopy (EDS) [11,16], IR and Raman spectroscopy [13,18,19,20,21], adsorption capacity [22,23], and so on [17,24,25]. IC can be used to determine the concentrations of specific ions or minerals. ICP provides quantitative information on trace and major elements in various sample types. XRD is used to identify the crystalline structure of minerals and helps to identify specific minerals that may be present in seawater concentrates. EDS is often used in conjunction with SEM to obtain information on the size, shape, composition, and chemical composition of mineral particles. IR and Raman spectroscopy, which complement each other, provide information about the molecular structure of minerals.

Alhimali et al. [11] investigated ion transportation in a microbial desalination cell (MDC) using IC, flame photometry, atomic absorption spectrometry, and EDS. Mourad et al. [13] identified solid products (e.g., NaHCO_3_, KCl, KHCO_3_, and Mg(OH)_2_, etc.) collected from a multi-stage flash desalination unit in the United Arab Emirates (UAE) using SEM, XRD, FTIR, and Raman spectroscopy to improve processes that can reduce brine salinity after desalination. Kress et al. [15] analyzed the quality of desalinated seawater in Israel using ICP to monitor the marine environment. Xue et al. [16] analyzed the shape and size of mineral particles obtained from seawater reverse osmosis (SWRO) desalination using EDS and XRD to detect pollution sources caused by SWRO desalination. Wang et al. [17] evaluated the performance of nanofiltration (NF) membranes using SEM images and permeation flux and rejection measurements.

To obtain a comprehensive view of mineral identification, it is ideal to apply all the above-mentioned technical methods. However, it is not easy to use all of them and it can be challenging to directly combine the information obtained from different methods on the same sample. Therefore, in this study, conventional methods such as SEM, EDS, XRD, IR, and Raman spectroscopy, along with the innovative approach of two-dimensional correlation spectroscopy (2D-COS), are applied to identify and provide complementary information about the mineral composition and molecular structure of the mineral extracts obtained during the desalination process. By combining conventional techniques with 2D correlation spectroscopy, the objective of this research is to overcome the challenges of integrating data from different analyses and provide a more holistic view of the mineralogical complexity of desalination extraction minerals.

Two-dimensional correlation spectroscopy [26,27] is a widely recognized method for analyzing spectra. In 1993, Noda [26] generalized this method to a wide range of applications. This method is applicable to a variety of systems, external perturbations, and spectroscopies, including chromatography techniques [26,27]. There are many advantages of 2D-COS in the interpretation of spectral data, including the determination of inter- or intramolecular interactions, the identification of the origin of each corresponding component, and the correlation of information from different spectroscopic sources through heterospectral correlation. It is particularly useful for studying time-dependent processes, such as chemical reactions, and for identifying spectral components that overlap in traditional one-dimensional spectra. It can provide insights into the kinetics, mechanisms, and interactions within a system. Additionally, 2D-COS can provide in-depth insights into the chemical processes occurring during desalination, mineral identification, and the optimization of desalination processes. Therefore, it enables a more comprehensive understanding of the chemical transformations and reactions that take place, thereby improving the efficiency, product quality, and environmental sustainability of the desalination process.

In this study, seawater extracts obtained in each desalination step are investigated using EDS, XRD, IR, Raman spectroscopy, and 2D-COS. This is the first study to apply 2D-COS to a desalination process system. From EDS, XRD, IR, and Raman spectroscopy, the composition species of seawater extracts are identified. Applying 2D-COS to XRD patterns, IR, and Raman spectra of seawater extracts at each desalination step provides information on the relationship between extracts obtained during the desalination process and the sequential order of intensity changes in each corresponding band. In addition, the presence of MgCl_2_(H_2_O)x in the Raman spectra, which cannot be captured in the 1D Raman spectra alone, can be clearly detected using heterospectral 2D XRD-Raman correlation analysis.

## 2. Results and Discussion

### 2.1. Identification of the Extracted Mineral Composition during the Desalination Process

#### Ions and Compounds of Each Extract

**Figure 1:**
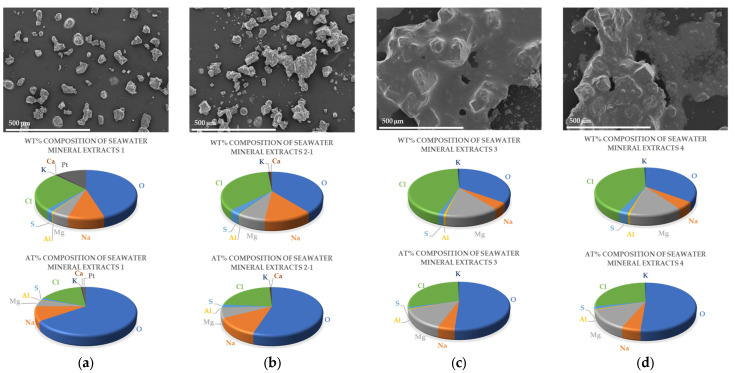
SEM images (top) and wt% (middle) and at% (bottom) composition graphs of minerals extracted from each desalination step: (**a**) first extracts; (**b**) second extracts; (**c**) third extracts; (**d**) final extracts.

In the SEM images of mineral extracts from East Sea water in Korea shown at the top of Figure 1, the first and second extracts look like separated particles, while the third and final extracts look like aggregated particles. The composition ratios, expressed as both weight and atomic percentages (wt% and at%, respectively), obtained from EDS analysis, are presented in the middle and bottom rows of Figure 1. The amounts of extracted mineral composites are detailed in Table 1. As shown in the middle and bottom rows of Figure 1 and in Table 1, each extract is typically composed of sodium (Na^+^), potassium (K^+^), magnesium (Mg^2+^), aluminum (Al^3+^), chloride (Cl^−^), sulfate (SO_4_^2−^), and oxygen (O^2−^) ions. Calcium (Ca^2+^) ions are present only in the first and second extracts. When seawater undergoes desalination, the amounts of Na^+^ and O^2−^ ions decrease, while the amounts of Mg^2+^ and Cl^−^ ions increase. Despite the decrease in the amount of Na^+^ and O^2−^ ions, the percentage of Na^+^ and O^2−^ ions in the extracted minerals is greater than 40 wt% (56 at%). This observation suggests that these ions are selectively retained in the minerals during the desalination process due to selective precipitation, ion exclusion or inclusion, ion competition, etc. [28,29].

The EDS analysis identified seven different mineral species: calcium chloride (CaCl_2_), sodium chloride (NaCl), calcium sulfate (CaSO_4_), potassium chloride (KCl), aluminum chloride oxide (AlOCl), magnesium chloride (MgCl_2_), and magnesium sulfate (MgSO_4_). The EDS results show that NaCl salts are the most abundant compounds in the first and second extracts, and MgSO_4_ and MgCl_2_ are present in the third and final extracts. To understand and identify these specific minerals, more detailed mineralogical and chemical analyses are needed.

**Figure 2:**
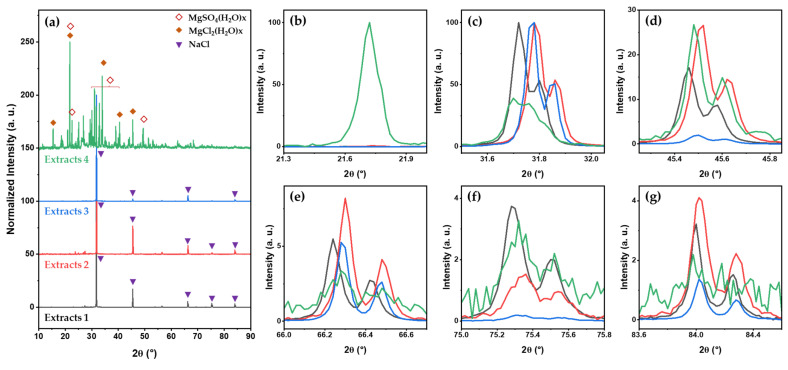
(**a**) XRD patterns of minerals extracted from each desalination step and magnified XRD patterns between diffraction angles of (**b**) 21.3° and 22.0°, (**c**) 31.5° and 32.1°, (**d**) 45.2° and 45.9°, (**e**) 66.0° and 66.7°, (**f**) 75.0° and 75.8°, and (**g**) 83.6° and 84.5°.

Figure 2 shows the XRD patterns of minerals extracted from each desalination step. The typical XRD patterns of NaCl [30,31,32,33] are observed in the first to the third extracts (see Figure 2a). There are five peaks indexed as (200), (220), (400), (420), and (422) at 31.7, 45.5, 66.3, 75.3, and 84.0°, respectively. Based on the XRD patterns, the lattice size of a NaCl crystal is calculated to be approximately 0.56 nm × 0.56 nm × 0.56 nm. In the magnified XRD patterns (see Figure 2c–g), the relative intensities and positions of each peak are different, indicating that there are traces of other minerals [34,35]. In the final extracts, XRD patterns corresponding to MgSO_4_(H_2_O)x and MgCl_2_(H_2_O)x are observed (see Figure 2a,b). The intensity and position of XRD patterns can change with the degree of mineral hydration [36,37,38,39,40]. In this study, the amount of H_2_O(x) cannot be determined from the XRD results alone. However, it is clear that MgSO_4_(H_2_O)x and MgCl_2_(H_2_O)x are not anhydrides (i.e., x ≠ 0), because the XRD patterns of anhydrides [36,37,40] are completely different from the results of this study. Moreover, anhydrous MgSO_4_ and MgCl_2_ are not stable under ambient conditions [41,42].

In general, the main ions in seawater minerals are Na^+^, Mg^2+^, Ca^2+^, K^+^, Cl^−^, and SO_4_^2−^ [43,44]. In this study, the main ions extracted in the desalination process are Na^+^, Mg^2+^, Cl^−^, and SO_4_^2−^. Based on the EDS and XRD results, it is assumed that the main components of East Sea water in Korea are NaCl, MgCl_2_, and MgSO_4_, or that these components can be easily extracted by desalination plants. The characterization and identification of specific minerals extracted during the desalination process were carried out using IR and Raman spectroscopy.

**Figure 3:**
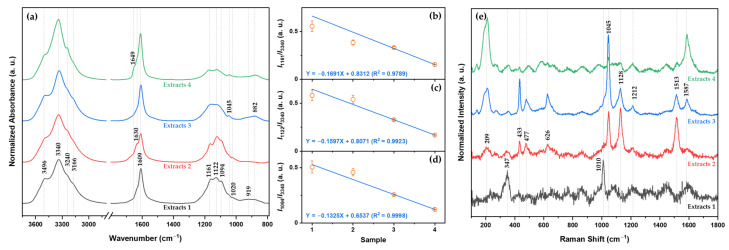
(**a**) IR spectra of minerals extracted from each desalination step. The relative intensity changes in the bands at (**b**) 1161, (**c**) 1122, and (**d**) 1094 cm^−1^ with respect to the band at 3340 cm^−1^ for each extract. (**e**) Raman spectra of the minerals extracted from each desalination step.

The IR spectra of minerals extracted from each desalination step are displayed in Figure 3a. There are characteristic bands of hydrated minerals associated with SO_4_^2−^. In the first extracts, 12 bands appear: the four bands at 3496, 3340, 3240, and 3166 cm^−1^ are the O-H stretching vibration mode; the band at 1609 cm^−1^ and the shoulder at 1630 cm^−1^ are assigned to the H_2_O bending mode; and the five bands at 1161, 1122, 1094, 1045, 1020, and 919 cm^−1^ are assigned to SO_4_^2−^ [45,46]. Based on the EDS analysis, XRD patterns, and some references [45,46], it is observed that the IR spectra correspond to MgSO_4_(H_2_O)x. There are no IR bands corresponding to NaCl and MgCl_2_(H_2_O)x [47,48]. When seawater undergoes desalination, all band shapes change. These changes correlate with changes in the degree of hydration of MgSO_4_(H_2_O)x. Figure 3b–d shows the relative intensities of the bands at 1161, 1122, and 1094 cm^−1^ relative to the band at 3340 cm^−1^ along the extract of each desalination step. The calibration curves are obtained using linear fitting (Y = A + BX equation) with parameters at a 95% confidence level. As shown in Figure 3b–d, the relative intensities of the mineral bands related to SO_4_^2−^ are linearly related for different extracts: for I_1161/3340_, Y = −0.1691X + 0.8312 (R^2^ = 0.9789); for I_1122/3340_, Y = −0.1597X + 0.8071 (R^2^ = 0.9923); and for I_1094/3340_, Y = −0.1325X + 0.6537 (R^2^ = 0.9998). This observation indicates that the quantitative analysis of the degree of hydration of MgSO_4_(H_2_O)x in each extract is possible using IR spectra.

The Raman spectra of the minerals extracted from each desalination step are shown in Figure 3e. In the first extracts, there are two main bands at 347 and 1010 cm^−1^ corresponding to CaSO_4_(H_2_O)x [49,50]. In the second and third extracts, there are ten bands at 209, 347, 433, 477, 626, 1045, 1128, 1212, 1513, and 1587 cm^−1^. Among them, the five bands at 626, 1045, 1128, 1212, and 1513 cm^−1^ correspond to MgSO_4_(H_2_O)x [51,52]. In the final extracts, the four bands at 209, 1045, 1513, and 1587 cm^−1^ remain. The two bands at 209 and 1587 cm^−1^ increase as seawater undergoes desalination. The band at 209 cm^−1^ is assigned to lattice vibrations [53]. The Raman spectra of the final extract are related to MgCl_2_(H_2_O)x. In addition, the XRD patterns of the final extract show the presence of MgSO_4_(H_2_O)x and MgCl_2_(H_2_O)x because the intensity of the band of MgSO_4_(H_2_O)x decreases.

### 2.2. Two-Dimensional Correlation Spectroscopy Analysis of Minerals Extracted from Each Desalination Step

The presence of specific minerals in the extracts obtained from the desalination process was confirmed using EDS, XRD patterns, and IR and Raman spectra. To analyze how the intensity changes in specific mineral (MgSO_4_(H_2_O)x) bands change during the desalination process, 2D-COS was applied to the IR spectra of minerals extracted from each desalination step. Furthermore, the application of heterospectral 2D-COS to XRD and Raman spectra provides comprehensive information on the mineral composition and molecular structure of the extracts.

**Figure 4:**
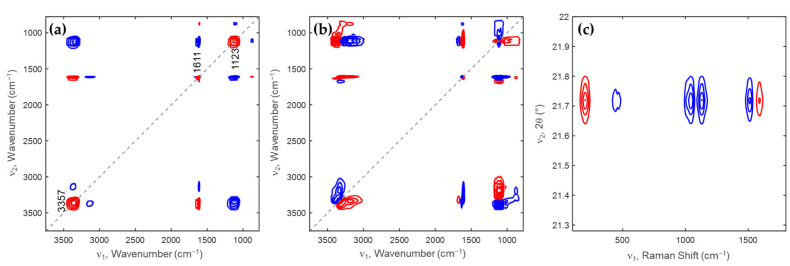
(**a**) Synchronous and (**b**) asynchronous 2D IR correlation spectra. (**c**) Synchronous 2D heterospectral XRD-Raman correlation spectra for the XRD pattern at 21.5–21.9° diffraction angles and Raman spectra in 100–1800 cm^−1^ spectral region. Positive and negative cross peaks, respectively, are red and blue lines.

Figure 4a,b display the synchronous and asynchronous 2D IR correlation spectra obtained from Figure 3a. As shown in Figure 1a, three autopeaks appear at 3357, 1611, and 1123 cm^−1^, indicating that significant changes occur in the hydration degree bands of the minerals related to SO_4_^2−^ as seawater undergoes desalination. There are two positive cross peaks at (1611, 3357) and (1611, 877) cm^−1^, indicating an increase in the bands at 877, 1611, and 3357 cm^−1^. There are five negative cross peaks at (3155, 3357), (1123, 3357), (1611, 3155), (1123, 1611), and (877, 1123) cm^−1^, suggesting a decrease in the bands at 3155 and 1123 cm^−1^. Based on this observation, the band at 3357 and 3155 cm^−1^ can be assigned to H_2_O dissociated from MgSO_4_(H_2_O)x, and H_2_O hydrogen-bonded to MgSO_4_, respectively. As shown in Figure 4b, the band at 1123 cm^−1^ observed in the synchronous spectrum is split into three bands at 1159, 1123, and 1092 cm^−1^. By comparing the sign of the cross peaks in synchronous and asynchronous spectra, the sequential order of the intensity changes of the bands can be determined: 1159 → 1092 → 1123 → 3357 → 3155 cm^−1^. This result means that nH_2_O dissociates from MgSO_4_(H_2_O)x to form MgSO_4_(H_2_O)x − n.

Figure 4c shows the synchronous 2D heterospectral XRD-Raman correlation spectra obtained from the XRD patterns in Figure 2b and Raman spectra in Figure 3e. The two bands at 203 and 1590 cm^−1^ in the Raman spectra are positively correlated with the XRD peak at 21.72° indexed to MgSO_4_(H_2_O)x and MgCl_2_(H_2_O)x. Interestingly, the band related to MgSO_4_(H_2_O)x is negatively correlated with the XRD peak at 21.72° indexed to MgSO_4_(H_2_O)x and MgCl_2_(H_2_O)x. Therefore, the two bands at 203 and 1590 cm^−1^ in the Raman spectra are certainly assigned to MgCl_2_(H_2_O)x.

In this study, the specific minerals NaCl, MgSO_4_(H_2_O)x, MgCl_2_(H_2_O)x, and CaSO_4_(H_2_O)x in the extracts obtained from the desalination process were identified and characterized using XRD, IR, Raman, and 2D-COS. In particular, the use of the heterospectral 2D-COS technique allowed for clear band identification.

## 3. Materials and Methods

The minerals extracted from each desalination step were sourced from the Korea Research Institute of Ships and Ocean Engineering (KRISO). All extracted minerals were placed in a vacuum oven for 24 h at room temperature and then ground to obtain FE-SEM images, EDS data, XRD patterns, and IR and Raman spectra. All extracted minerals were also ground for XRD patterns, IR spectra, and Raman spectra measurements.

FE-SEM images and EDS data were obtained using a JSM-7900F (JEOL, Hwaseong-si, Republic of Korea) with a lower electron (LE) detector, 10.0 kV accelerating voltage, and ×120 magnification. EDS data were collected for particles in a 1 × 1 mm^2^ area. XRD patterns were measured using a D8 Discover+ X-ray diffractometer (Bruker, Mannheim, Germany) with a Cu Kα X-ray source (λ = 1.5406 Å). IR spectra were measured using a Nicolet iS50 FTIR Spectrometer (Thermo Fisher Scientific Inc., Waltham, MA, USA) with a built-in attenuated total reflectance (ATR, diamond crystal, 45°) and a deuterated lanthanum triglycine sulfate (DLaTGS) detector. Each IR spectrum was measured 3 times with 4 cm^−1^ spectral resolution and 32 scans. Raman spectra were collected using a LabRAM HR-evolution Raman spectrometer (Horiba, Tokyo, Japan) with a SynapsePlus OE scientific CCD camera (air-cooled: −75 °C). The Raman shifts were calibrated using a silicon wafer exhibiting a well-known band at 520 cm^−1^. Each Raman spectrum was collected at least 3 times using a 532 nm laser with an exposure time of 2 s and 10 accumulations using a 532 nm laser.

All XRD patterns were baseline-corrected and then intensity-normalized using the maximum intensity peak of each pattern using the OriginPro 2022b program (OriginLab Corporation, Northampton, MA, USA). All IR and Raman spectra were also baseline-corrected and then area-normalized using Solo9.2 software (Eigenvector Research, Inc., Manson, MA, USA). IR and Raman spectra were then averaged using the OriginPro 2022b program. We analyzed the calibration curve between the relative intensities vs. each extraction of IR spectra using linear fitting (95% confidence level of parameters) with the OriginPro 2022b program. The 2D-COS analysis was performed with homemade code using MATLAB R2023a software (MathWorks, Inc., Natick, MA, USA). Two-dimensional correlation spectra are displayed as synchronous and asynchronous spectra. In the synchronous spectrum, autopeaks and cross peaks appear on the diagonal and off-diagonal lines, respectively. Autopeaks represent overall intensity changes due to external perturbations. Cross peaks indicate the direction of the intensity changes for two bands that are correlated with each other. A positive cross peak means that two bands are increasing or decreasing with external perturbation, while a negative cross peak indicates that one band is increasing and the other is decreasing with external perturbation. In the asynchronous spectrum, cross peaks appear only on off-diagonal lines. This improves spectral resolution because it reveals differences in response time or time delays between the two spectra. By comparing the sign of cross peaks in both the synchronous and the asynchronous spectra, the sequential order of the band intensity changes under external perturbation can be determined.

The synchronous 2D correlation spectrum is derived by multiplying matrix A by its transposed matrix. The asynchronous 2D correlation spectrum is induced by multiplying matrix A by the Hilbert–Noda transformation matrix and its transposed matrix. Here, matrix A represents the dynamic spectra obtained by subtracting the averaged spectra from the pretreated spectra (IR or Raman spectra) and is of size *m* × *n* (*m* is variables and *n* is perturbation). Hilbert–Noda transformation (N_jk_) is of size *m* × *m*, where the diagonal elements N_jk_ are all zero, and off-diagonal elements are given by N_jk_ = 1/{*π*(*k* − *j*)}. The 2D heterospectral XRD-Raman correlation spectrum is generated by multiplying the product of the matrix of XRD patterns (*m* × *n*) and the matrix of Raman spectra (*l* × *n*), where *m* and *l* are variables and *n* is perturbation. The book by Noda [27] is described in detail through the computation of 2D-COS.

## 4. Conclusions

In this study, the mineral components extracted from the desalination process were characterized using SEM, EDS, XRD, IR, and Raman spectroscopy. To further analyze the minerals extracted from each desalination step, 2D-COS was also applied to the XRD patterns, IR spectra, and Raman spectra. The EDS analysis and XRD patterns demonstrate the presence of minerals, such as NaCl, MgCl_2_, and MgSO_4_, extracted through the desalination process. The results of IR and Raman spectroscopy indicate that the hydration degree of SO_4_^2−^-related extracts decreases as seawater undergoes desalination. Furthermore, 2D-COS provides information about the changes in the extracts obtained from the first to final stage. In particular, the results of heterospectral XRD and Raman 2D-COS provide clear assignments in the Raman spectra. Notably, the first application of 2D-COS to the desalination process has helped us to better understand the properties of seawater extracts.

## Figures and Tables

**Table 1:** EDS analysis of mineral composites extracted from each desalination step.

	Sample	Extract 1	Extract 2	Extract 3	Extract 4
Atoms					
Oxygen (O)	Weight (%)	45.16	39.22	35	35.26
	Atomic (%)	65.38	54.97	51.12	51.29
Sodium (Na)	Weight (%)	9.61	12.69	4.43	5.02
	Atomic (%)	9.68	12.38	4.5	5.09
Magnesium (Mg)	Weight (%)	4.68	7.18	14.16	13.87
	Atomic (%)	4.46	6.62	13.61	13.28
Aluminium (Al)	Weight (%)	0.37	0.23	0.53	0.53
	Atomic (%)	0.32	0.19	0.46	0.46
Sulfur (S)	Weight (%)	1.83	3.22	1.44	2.51
	Atomic (%)	1.32	2.25	1.05	1.82
Chlorine (Cl)	Weight (%)	25.95	35.86	43.85	42.35
	Atomic (%)	16.96	22.68	28.9	27.8
Potassium (K)	Weight (%)	0.57	0.99	0.58	0.45
	Atomic (%)	0.34	0.57	0.35	0.27
Calcium (Ca)	Weight (%)	0.32	0.61	0	0
	Atomic (%)	0.19	0.34	0	0
Platinum (Pt)	Weight (%)	11.5	0	0	0
	Atomic (%)	1.37	0	0	0

## Data Availability

The data presented in this study are available on request from the corresponding author.
